# Technology for Healthy Aging and Wellbeing: Co-producing Solutions

**DOI:** 10.3389/fpsyg.2021.745947

**Published:** 2021-12-02

**Authors:** Arlene J. Astell, Jacob A. Andrews, Matthew R. Bennion, David Clayton

**Affiliations:** ^1^School of Psychology and Clinical Language Sciences, University of Reading, Reading, United Kingdom; ^2^KITE Research Institute, University Health Network, Toronto, ON, Canada; ^3^Department of Occupational Sciences & Occupational Therapy, University of Toronto, Toronto, ON, Canada; ^4^Department of Psychiatry, University of Toronto, Toronto, ON, Canada; ^5^NIHR Mindtech Medtech Co-operative, Mental Health and Clinical Neurosciences, University of Nottingham, Nottingham, United Kingdom; ^6^Department of Computer Science, University of Sheffield, Sheffield, United Kingdom; ^7^Department of Psychology, University of Sheffield, Sheffield, United Kingdom; ^8^Leicester School of Nursing and Midwifery, De Montfort University, Leicester, United Kingdom; ^9^Centre for Life Long Learning, Warwick University, Coventry, United Kingdom

**Keywords:** mental health, wellbeing, technology, aging, co-production, methods

## Abstract

Methods to facilitate co-production in mental health are important for engaging end users. As part of the Technology for Healthy Aging and Wellbeing (THAW) initiative we organized two interactive co-production workshops, to bring together older adults, health and social care professionals, non-governmental organizations, and researchers. In the first workshop, we used two activities: Technology Interaction and Scavenger Hunt, to explore the potential for different stakeholders to discuss late life mental health and existing technology. In the second workshop, we used Vignettes, Scavenger Hunt, and Invention Test to examine how older adults and other stakeholders might co-produce solutions to support mental wellbeing in later life using new and emerging technologies. In this paper, we share the interactive materials and activities and consider their value for co-production. Overall, the interactive methods were successful in engaging stakeholders with a broad range of technologies to support mental health and wellbeing and in co-producing ideas for how they could be leveraged and incorporated into older people’s lives and support services. We offer this example of using interactive methods to facilitate co-production to encourage greater involvement of older adults and other under-represented groups in co-producing mental health technologies and services.

## Introduction

Co-production or ‘making things together’ (Social Care Institute for Excellence [SCIE], 2015) is specifically encouraged in health and social care in the United Kingdom and other countries to engage service users. In the United Kingdom co-production was introduced as a key lesson from two public inquiries which identified that “service providers need to develop more equal partnerships with people who use services and carers” (Social Care Institute for Excellence [SCIE], 2015). Co-production is based on five principles: building and maintaining relationships, reciprocity, sharing of power, including all perspectives and skills, and respecting and valuing the knowledge of all (New Economics Foundation [NEF], 2013). Successful co-production requires an environment where everyone’s voice is heard, which is essential for populations not used to speaking in public, or with unequal power dynamics, such as patients speaking with doctors, or frontline staff speaking to managers and commissioners (Astell and Fels, 2021).

Co-production has been used in a variety of healthcare settings to improve services, increase choice, respond to user needs and reduce waste (Batalden et al., 2016). One aspect of co-production that can be difficult to achieve is power sharing (Farr et al., 2021). This is especially so in mental health research where it is a systemic barrier to engaging people with lived experience (Clark, 2015). Despite this obstacle, examples of co-production in mental health exist, including as a method to overcome health inequalities (Lwembe et al., 2017). People with lived experience of mental health problems have also been involved in co-producing programs including self-management of depression (Turner et al., 2015), and youth mental health (Mayer and McKenzie, 2017).

Fewer co-production studies have been conducted around late life mental health, in part due to negative perceptions about the abilities of older adults to participate (Mannheim et al., 2019). However, late life mental health is a growing global concern, with depression peaking in over 55s (World Health Organization [WHO], 2017). Co-production involving older adults in identifying gaps, priorities and developing new solutions is urgently needed. In particular, exploration of the potential of technology as part of services to support people’s mental health and wellbeing is increasingly important.

Fortunately, there is growing evidence that older adults are interested in exploring technology to self-support their mental health and wellbeing (Mannheim et al., 2019). However, there are still barriers to seeking mental health support, including fear of being labeled, difficulty navigating mental health services or mistrust of those working in these services (Reynolds et al., 2020). Older adults are also wary of who will have access to their data, how their data will be used, and of being a burden to others (Andrews et al., 2019). Additionally, older adults, especially those who are socially isolated and on fixed incomes, are more likely to be digitally excluded (Helsper and Reisdorf, 2017). These ‘hard to reach’ lonely and socially isolated individuals also have a significantly increased risk of depression (Lee et al., 2021). Exploring how stakeholders from different sectors, including older adults, view the potential of technologies in the support of mental health and how they can be implemented is therefore timely.

To explore understanding of technology for supporting late life mental health, we organized two co-production workshops using interactive tools as part of the Technology for Healthy Aging and Wellbeing (THAW) Ph.D. network. The THAW projects investigated three topics: (1) Using technology to improve early detection of late life mental health problems; (2) Understanding social isolation in a connected society; and (3) Affective computing to support good mental health in later life. Bringing together older adults, health and social care professionals, non-governmental organizations, and researchers, each workshop was facilitated with specific goals in mind. In designing these co-production workshops, the THAW team were aiming to gather information about how late life mental health and wellbeing were viewed and understood by attendees, attitudes to the role of technology in addressing late life mental health and wellbeing, and stakeholders’ visions for how emerging technologies could be utilized to benefit late life mental health and wellbeing. These facilitated workshops were also designed for attendees to have the opportunity to speak with people they did not normally come into contact with, make new contacts and have the opportunity to try out and provide feedback on existing and emerging technologies. Here, we describe the organization of the two workshops, the methods used and share the outcomes in respect of technology for late life mental health, to illustrate the utility and practicality of our approach for other researchers interested in co-production on this topic.

## Workshop One. ‘Mental Wellbeing: Can Technology Help Older People Lead a Healthier and Happier Life?’

### Aims and Objectives

The primary aim was to establish the feasibility of co-production for exploring the potential of new and emerging technologies in the support of older adults’ mental health. The secondary aim was to form a network of experts by experience who could contribute to future events and research activities.

### Design

This 4-h long workshop was designed to provide an inclusive environment where everyone’s voice could be heard. Two interactive activities – Technology Interaction (Astell et al., 2020) and Scavenger Hunt (Astell et al., 2020) – were combined with student presentations on the three THAW projects. Activity descriptions, materials and aims are set out in Table 1.

**TABLE 1:** Design of THAW interactive workshops.

**Activity breakdown**	**Description**	**Materials**	**Purpose**
**Workshop 1: ‘Mental well-being. Can technology help older people lead a healthier and happier life?’**			
Registration (15 min)	Attendees sign in and are assigned to a table	Sign in sheet, Information Sheet 1 and 2, name labels	Provide an inclusive environment to encourage sharing and collaboration.
Welcome and introduction (5 min)	Host welcomes attendees and explains the program	PA system	Introduction to the purpose and format of the workshop
Technology Interaction^a^ (70 min)	Working in pairs attendees choose an item from their technology mystery box and have 10 min to learn how to use it (Figure 1). Each pair shares their experience with the rest of their table and made notes on flipcharts. A representative from each table feeds back their experience to the whole room based on the Technology Interaction Feedback Sheet.	Thirty-six existing technologies with required power sources (batteries/mains power) six in each of six boxes, one per table (full list of items available from the corresponding author). Technology Interaction Feedback Sheet Flipcharts and marker pens	An icebreaker activity to foster interaction, collaboration, and give all participants confidence to speak about technology in the group setting.
Networking lunch (45 min)	Chat with other attendees and visit stalls	Stalls for providers of mental health support, including national charities, researchers, technology companies	Opportunity to speak with other attendees and visit stalls
THAW Ph.D. presentations (30 min)	Brief presentations of their work by THAW Ph.D. students	PowerPoint, projector, screen, microphone	To inform attendees about the current state of the three THAW projects
Scavenger Hunt^b^ (60 min)	Working in pairs each attendee tries out each device, app, or service and completes the Scavenger Hunt Feedback Form for each item.	Six stations with technologies relating to each of the three THAW projects (Table 2). Mr Mood and Lexulous were set up on tablets. MoodPanda and social networking sites on desktop touchscreen computers. MantaroBot telepresence robot plus laptop for operating. Scavenger Hunt Feedback Form.	For attendees to try out a range of existing and emerging technologies and provide feedback.
Tea and evaluation (15 min)	Eat, drink and complete evaluation	Workshop Evaluation Form Drinks and cake	Provide feedback to THAW team and final networking opportunities
**Workshop 2: ‘Healthy aging within your reach: Shaping new technologies to support mental wellbeing for older people’**			
Welcome and Housekeeping (10 min)	Welcome to the workshop and program	Information Sheet 1 and 2, name labels	Introduce the activities and house rules
Vignettes^c^ (60 min)	Each participant reads their vignette and takes notes before discussing as a group. Discussion has two parts: (1). Explore situations that present risks for developing mental health problems, loneliness and social isolation. (2). Explore different stakeholder perspectives or partner or other family member, Commissioner of service, GP, ICT developer, researcher (Supplementary Appendix A)	Six vignettes presenting an individual in a situation that could lead to loneliness, social isolation or have a negative impact on mental wellbeing (Supplementary Appendix A). The vignettes were written in the first person, enabling participants to draw on their personal experiences whilst avoiding personal disclosure, protecting their confidentiality and allowing discussion of sensitive and complex issues safely. Each vignette was different for each table but contained a similar amount of information and included descriptions such as age, health and family relationships and was accompanied by questions designed to gather information from the participants. Pens, paper and post-it notes.	To gain insights into the views, feelings and interpretations of the attendees about late life mental health and the perspectives of different stakeholders.
Refreshment break (20 min)	N/A	N/A	N/A
Scavenger Hunt (30 min)	Attendees invited to try six technologies in turn, scoring them on the abridged SUS plus additional comments about each technology’s potential application in health and care.	Six technology stations: ‘Pacifica,’ ‘Eliza,’ ‘MiRo,’ ‘MantaraBot,’ ‘Augmented Reality’ and ‘HTC Vive Virtual Reality.’ Abridged SUS^d^	For attendees to try out a range of existing and emerging technologies and provide feedback.
Networking lunch (45 min)	As workshop 1		
THAW Ph.D. presentations (45 min)	Early detection of mental health problems (15 min) Tackling social isolation (15 min) Avatars for older adults (15 min)	PowerPoint, projector, screen, microphone	To inform attendees about the current state of the three THAW projects
Invention Test^e^- Co-creating a new product or service (45 min)	Each group was invited to design a new technology-based tool or service, inspired by the vignettes they had considered in Vignettes and the technologies they had experienced in Scavenger Hunt.	Flipchart, paper and pens	To explore the impact on thinking about late life mental health, of the Vignettes and technologies introduced in the Scavenger Hunt
Presenting solutions (30 min)	Each group briefly presented their solution to the rest of the attendees	n/a	To see result of co-production activities
Tea and evaluation (15 min)	Complete evaluation	Workshop Evaluation Form Drinks and cake	Provide feedback to THAW team and final networking opportunities

*^*a*^ McGrath et al. (2016).*

*^*b*^ Astell et al. (2020).*

*^*c*^ Kandemir and Budd (2018).*

*^*d*^ Bennion et al. (2020). ^*e*^ McGrath et al. (2016).*

### Attendees

A mapping exercise was undertaken to identify local older people and carer’s groups/forums and organizations who may wish to be involved. Information was put on the Centre for Assistive Technology and Connected Healthcare (CATCH; University of Sheffield) website and flyers were produced to promote the workshop to stakeholders by email and social media. To secure the widest participation, the following stakeholders with an interest in the mental wellbeing of older people were invited: older people and informal carers; local Councils – social care practitioners and commissioners; Health Authorities – mental health practitioners and commissioners; third sector providers; researchers. Thirty-two people registered and 36 turned up on the day.

### Materials

Materials for the workshop were as follows: Six tables with six chairs at each for attendees. Box of six small technologies on each table. Five tables around the sides of the room for Scavenger Hunt. Refreshment station, PA system, and digital camera.

Information Sheet 1: Running order of activities/program for the day.

Information Sheet 2: Ground rules to ensure an inclusive, safe, respectful, and positive event

Stalls were set up in an annex for local organizations to promote their activities relating to late life mental health and wellbeing.

Technology Interaction: Feedback Sheet for collecting the following information: ‘What is the technology?,’ ‘How do you feel using it?,’ ‘What worked and/or didn’t work well with the technology?’ e.g., any good and bad points, ‘Who do you feel could use it?’ and ‘Do you think this could support or hinder mental wellbeing and make people healthier or happier?’

Scavenger Hunt: Six technology stations around the room, and Feedback Form with six boxes, one per item, with the following statements: ‘This is easy to use,’ ‘This is something I would use,’ ‘This is something I enjoy,’ with a space to circle ‘yes’ or ‘no.’ Names and descriptions of the specific technologies are detailed in Table 2. These were chosen for their connection to each of the THAW projects (see Table 2).

**TABLE 2:** Technological apps/equipment used for Workshop 1 and Workshop 2 Scavenger Hunts.

**Technology**	**Website**	**Description**	**THAW project**
**Workshop 1 Scavenger Hunt**			
MoodPanda	https://moodpanda.com	Mood tracking app, website and community	Project One: Using technology to improve early detection of mental health problems
Mr Mood	N/A – App store	Mood tracking app	
Lexulous (scrabble)	https://www.lexulous.com	Online word game	Project Two: Understanding social isolation in a connected society
Social networking sites	Senior Chatters: https://seniorchatters.co.uk	Chatrooms for over 50s	
	Side by Side: https://sidebyside.mind.org.uk	Online community of United Kingdom mental health charity mind	
	Buzz50: https://www.buzz50.com	Chatrooms and social networking for over 50s	
	Silver Surfers: https://www.silversurfers.com	Community for seniors	
	Giving and getting: http://www.nottscf.org.uk	Website for connecting people run by Nottinghamshire Community Foundation	
MantaroBot (broadcaster)	http://www.mantarobot.com/		Project Three: Affective computing to support good mental health in later life
MantaroBot (receiver)	http://www.mantarobot.com/		
**Workshop 2 Scavenger Hunt**			
Pacifica app	https://www.good-thinking.uk/resources/pacifica/	App which supports users experiencing stress, anxiety or depression through aspects	
		of cognitive behavioral therapy, mindfulness and meditation.	
Eliza	N/A	Eliza is a conversational agent designed in the 1960s which uses basic natural language	
		processing to provide responses to user-inputted text.	
MiRo (Robot dog)	http://consequentialrobotics.com/	MiRo is described as a programmable, autonomous robot, with moving head, ears and eyes.	
		MiRo moves on wheels. The robot has form features of a dog and a rabbit.	
MantaroBot	http://www.mantarobot.com/	MantaroBot is a telepresence robot consisting of a base motor unit on wheels with a metal	
		frame providing support for a tablet computer, enabling interaction and viewing by a	
		standing adult. The motor base unit can be controlled remotely via wifi.	
Augmented reality (AR)	n/a	A tablet computer with augmented reality software was used to demonstrate the potential of	
		the technology. It was demonstrated that AR could be used in relation to medication	
		management using a scan of medication labels to generate specific user instructions.	
HTC Vive Virtual Reality	https://www.vive.com/	An HTC Vive headset was used to demonstrate the potential of virtual reality.	

Workshop Evaluation Form.

### Procedure

**FIGURE 1:**
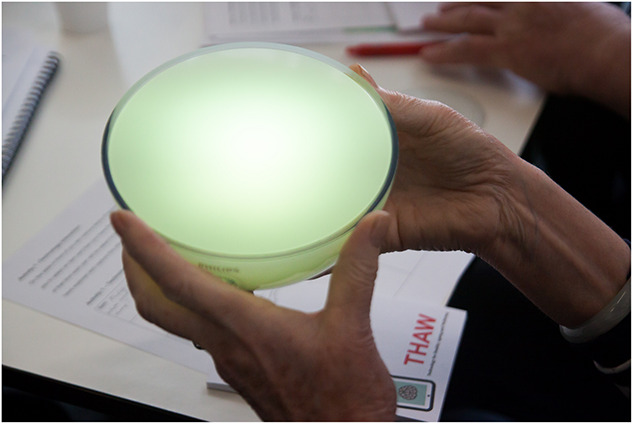
Mood light.

Attendees signed in, were assigned to the five tables, and asked to introduce themselves. Information Sheets 1 and 2 were provided to each participant, and they were asked to respect the equipment, not to use it unsafely or put others at risk, let everyone have a go and have a say and to treat other people with respect. After an introduction to the aims of THAW, attendees engaged in Technology Interaction (Table 1 and Supplementary Appendix A). This required pairs of attendees to try to get a piece of technology working in 10 min (Figure 1). A wide range of different technology types were provided to stimulate initial discussions and break the ice.

After a networking lunch, attendees had the opportunity to visit various stalls and speak with researchers, technology companies, and providers of mental health support, including a number of national charities. This was intended to provide further opportunities for attendees to find out about and discuss local services and support. The afternoon session resumed with brief presentations on each of the THAW doctoral projects. The presentations outlined the three different research projects and enabled attendees to gain an insight into the different elements being explored. Following these presentations, attendees engaged in the second interactive activity: Scavenger Hunt as described above (Table 1). The workshop ended with further networking opportunities over afternoon tea, where attendees were asked to complete the Workshop Evaluation Form before they left.

### Results

The 36 people who attended the workshop were: 13 health or social care staff, 11 older adults, 4 advocates for older adults, 3 researchers and 5 others, comprising 1 commissioning manager of public health, 1 health and social care commissioner, 1 charity project worker, 1 volunteer with older people and 1 activities coordinator. Information about age or sex/gender was not collected from attendees.

#### Technology Interaction

The aim of Technology Interaction is to empower attendees to feel confident and comfortable discussing technology. It also promotes discussion of what people like and dislike in a range of technologies. Feedback from the five groups (one per table) identified features of the technologies that they liked, and the ones they did not like among the selection of off-the-shelf technologies. These features influence people’s reaction to a device and whether they will persevere with trying to use it. Positive features included items being simple or intuitive to use, items that have clear accessible instructions, and items where the intended use is obvious. For example, a well-received TomTom navigation device was described as “very easy and logical, it could be used by anyone, not just drivers.” Negative feedback highlighted lack of instructions, small text or small buttons, lack of indication what exactly the technology is for or how it should be used. A blood pressure cuff was rejected because it was “okay – comes with a manual but no idea what to expect. Took three attempts to get all readings and mine was not accurate.” During the feedback component, one person commented that it was clear that services could not or should not just give devices to people to work out for themselves.

#### Scavenger Hunt

Attendees’ answers on the feedback forms to the three use questions about each of the six technologies (Table 2; MantaroBot was presented as two technologies – one as controller and one as receiver), showed clear differences in their perceptions. Of the six technologies the two mood apps (MoodPanda and Mr Mood) and social networking sites were judged most easy to use and the MantaroBot in both roles (as controller and receiver) as least (Table 2). Despite the low rating of its usability, attendees saw potential in the MantaroBot: “Provides digital telepresence for engagement and inclusion” and “I feel the MantaroBot is a useful tool and would be able to support/navigate face to face contact with an individual.”

Of particular interest, while the two mood apps were judged easy to use, the majority of attendees who completed the feedback say they would neither use nor enjoy these apps. Their comments indicated concerns about the age appropriateness of the apps “Simple, childlike”; the functionality “useful to record mood over time but very limited”; and usefulness: “Does it add to a paper diary?” By contrast, attendees found the social networking sites easy to use and the majority said they would enjoy using them: “…Allows access to a wide range of support.” Similarly, Lexulous, an online scrabble game was rated in the middle for ease of use, but more people would use it and enjoy it than any of the other technologies. However, this did seem to depend on whether people were already digital gamers.

### Workshop Evaluation

The workshop activities appeared successful in encouraging attendees to work together and share their expertise and experience. As one attendee commented…

“I felt that the group work at the beginning [Technology Interaction] worked really well as it allowed people from different generations to work out how to use bits of technology that they may have never seen before. It allowed people to share ideas, views and opinions on use, ease, and product design.”

Many forms commented on the diversity of professions and representatives among attendees, for example:

“meeting people from a broad range of organizations, community groups, third sector” and “interacting with people from different backgrounds and experience in social and health care”

For one attendee:

“by talking to lots of people” [they got] “a stronger feeling that technology will help people now and, in the future.”

While another was impressed with

“how the event was set up and enabled more effective networking.”

The mix of activities and presentations was also welcomed by attendees with one attendee commenting on the importance of “being able to see the technology and hear about research” in the THAW Ph.D. presentations.

At the end of the day, the majority of attendees agreed that the workshop had helped them gain a better understanding of the needs, barriers, and benefits of technology for mental wellbeing in later life. One attendee was:

“excited about technology and how much it’s changing in relation to older adults and mental health.”

Another felt the workshop was:

“thought-provoking, some very clever ideas that could be adopted to improve the lives of older people.”

One attendee also proposed the interactive aspect of the workshop could have a therapeutic value to those with mental health issues:

“Not just about today but as a former volunteer tutor for the Expert Patients’ Program and with relatives who work in mental health, I would like to stress the vital importance of enabling people to interact and offer mutual support in coping with their problems.”

### Workshop 1 Summary and Reflection

Overall, the first workshop was a success in attracting a diverse range of attendees collaborating together to gain insight and interest in mental wellbeing and technology helping older people lead a healthier and happier life. The Technology Interaction activity successfully created a supportive environment for attendees to interact and share their views of technology. In terms of feedback, attendees identified factors that influence their decisions about persevering with or rejecting a technology, highlighting instructions, ease of use and functionality as key features. The Scavenger Hunt revealed the differing impressions and assessments of the six technologies. There was some suggestion that familiarity influenced the judgment about ease of use but did not influence people’s judgments about using and enjoying the technologies.

The findings confirmed that older adults could engage as equals in discussions about new and emerging technologies. They were interested to try the technologies on offer and were able to share their opinions and responses to them. The older adults were also comfortable discussing late life mental health and tools for supporting this. The interactive activities and workshop format were also successful at facilitating participation by frontline staff in health and social care with older adults, researchers, and other stakeholders. Encouraged by these results we organized a second workshop to focus on co-producing new solutions for late-life mental health and wellbeing.

## Workshop Two: ‘Healthy Aging Within Your Reach: Shaping New Technologies to Support Mental Wellbeing for Older People’

### Aims and Objectives

The second THAW workshop focused on co-production of solutions involving technology for late-life mental health and wellbeing.

### Design

Building on the success and lessons learnt from the first workshop, activities were designed to facilitate discussion and thinking about ways of using new technology to support mental wellbeing in later life within services. This second workshop lasted 5.5 h and included three interactive activities: Vignettes (McGrath et al., 2016), Scavenger Hunt (Astell et al., 2020), and Invention Test (McGrath et al., 2016). These are detailed in Table 2.

### Attendees

A list of invitees was created from the attendees at the first workshop plus other technology workshops hosted by CATCH who expressed an interest in attending future events. As before, information was put on the CATCH Website and flyers were produced to promote the workshops to stakeholders by email and social media. A total of 48 attendees registered and on the day 50 people attended – demographic data was not collected and not all attendees supplied their affiliation or role.

### Materials

Materials for the workshop were as follows: Six tables with eight/nine chairs at each for attendees. Five tables around the sides of the room (for Scavenger Hunt). Refreshment station, PA system, and digital camera.

Information Sheet 1: Running order of activities/program for the day.

Information Sheet 2: Ground rules to ensure an inclusive, safe, respectful, and positive event.

Stalls for local organizations to promote their activities relating to late life mental health and wellbeing.

Vignettes: Six vignettes presenting an individual in a situation that could lead to loneliness, social isolation or have a negative impact on mental wellbeing. The process of creation and content for the vignettes used personal insights and professional experiences of working with older people from all the THAW researchers. They were written to enable participants to reread the scenario and discuss it on their table. Each vignette was different for each table but contained a similar amount of information relating to gender, age, presenting health conditions, family relationships, possible triggers or threats to social isolation, loneliness and mental well-being, and digital technological use. It was felt important that the vignette should resonate with participants (Skilling and Stylianides, 2020) and so the vignettes were written in the first person enabling participants to draw on their personal experiences whilst avoiding personal disclosure, protecting their confidentiality, and allowing discussion of sensitive and complex issues safely. As the range of stakeholders attending was diverse, the terminology used in the vignettes avoided jargon and was kept simple. The vignettes included six different perspectives; the characters involved in the scenario along with characters who may support and shape new services and the use of new technologies to help the mental well-being of older people (Table 1 and full details in Supplementary Appendix A).

Scavenger Hunt Materials: Six technology stations, detailed in Table 2. These were chosen as emerging technologies which may have a role in future health and care services.

Abridged version of the System Usability Scale (SUS; Brooke, 1996) which measures perceived usability – as used in other older adult studies relating to THAW (Bennion et al., 2020). A SUS score above 68 represents an above-average usability (Brooke, 2013).

Invention Test: Flipchart, paper, pens.

Workshop Evaluation Form.

### Procedure

As Workshop 1, attendees signed in, were assigned to tables, and asked to introduce themselves. Information sheets 1 and 2 were given to each attendee asking them to respect the equipment, not to use it unsafely or put others at risk, let everyone have a go and have a say and to treat other people with respect. After an introduction to the aims of the day, attendees engaged in activity 1, Vignettes (Table 1). Following a 15-min refreshment break, attendees engaged in the second interactive activity: Scavenger Hunt (Table 1) with six new and in development technologies (Table 2). This included a new version of Eliza, the original therapy chatbot, developed as part of the THAW project studying the potential of chatbots in therapeutic interventions for older adults.

After a networking lunch and visits to stalls, the afternoon session resumed with brief update presentations on each of the THAW doctoral projects (Table 1). Following these presentations, attendees completed the Invention Test (Table 1). A representative from each group gave an overview of their idea to the whole workshop attendance at the end of the day and a prize was awarded to the best invention. The overviews were filmed and analyzed using Content Analysis. The workshop ended with further networking opportunities over afternoon tea, where attendees were asked to complete the Workshop Evaluation Form before they left.

### Results

#### Vignettes

Each group of stakeholders were provided with one written vignette depicting a snapshot of a situation for an older person where mental wellbeing was an issue. The vignettes were used to introduce a range of factors and situations that could negatively impact or threaten late life mental health and wellbeing. The groups were asked to discuss these different perspectives. The vignette activity successfully stimulated discussion within the groups and fostered thinking about the need for and potential of holistic solutions. This discussion also set the scene for the other two activities. Participants engaged with, discussed, and identified the factors that affect mental well-being in later life in their vignettes. At the end of the workshop, the majority of attendees reported that they had a better understanding of the factors that could contribute to good and poor mental health and a better understanding of the roles of different services and organizations that can support mental health in later life. Participants appreciated the contribution of the vignettes to this improved understanding. One person, for example, valued: “Working together as a group to look at how we can deal with our given case study.” While another valued: “Exchanging views and ideas of how best to support the person in our case study”

The vignettes were referred to throughout the day. In particular, the combination of bringing together stakeholders with current and emerging technology, and a vignette to shape the discussions, created an environment where new ideas and thinking could be generated. Overall, 97% of attendees agreed or strongly agreed that the event had generated new thinking and a number of groups worked together and were inspired by the vignette personas when developing their ideas in the last activity of the day, the Invention Test (see below).

#### Scavenger Hunt

In total, 43 attendees completed the abridged SUS questionnaire. The sample was comprised of 39.5% (17/43) Health/social care staff, 25.6% (11/43) Older adults, 4.7% (2/43) Academic and Research staff, 7.0% (4/43) Policy Makers, 7.0% (4/43) Industry, Advocate for Older Adults or Others and 16.3% (7/43) Undisclosed.

**FIGURE 2:**
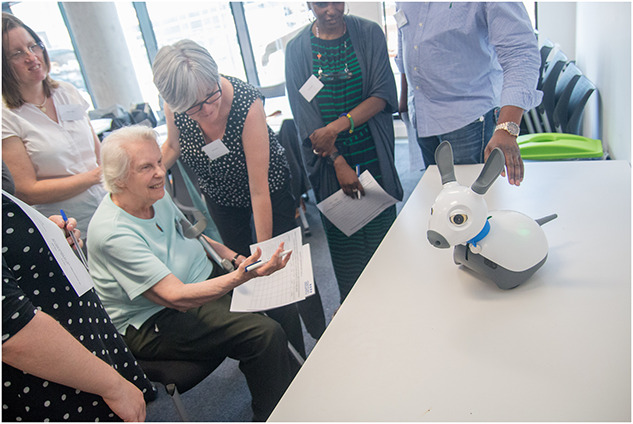
MiRo.

Across all groups only two of the six technologies had a SUS score that was equal to or above the acceptable cut-off point (i.e., >68) (see Table 3): these were ‘MiRo’ (Figure 2) with a mean of 82.96 (SD 16.74) and ‘Pacifica’ with a mean of 68.37 (SD 21.73). Looking just at the older adults only MiRo scored above the SUS cut-off (mean of 81.25 [12.59]; see Table 3), while health and social care staff rated both MiRo (mean of 89.34 [16.01]) and Pacifica (mean of 76.84 [SD 17.83]) as usable.

**TABLE 3:** Mean (SD) for System Usability Scale measure.

**Technology (*n*)**	**SUS, mean (SD)**
Pacifica app (*n* = 41)	68.37 (21.73)
Eliza (*n* = 38)	56.25 (20.24)
MiRo (*n* = 42)	82.96 (16.74)
MantaroBot (*n* = 41)	38.61 (21.85)
Augmented reality (*n* = 37)	54.22 (21.03)
HTC Vive Virtual Reality (*n* = 38)	51.32 (20.09)

The SUS scores provided an informal ranking of the usability of the six technologies with MiRo highest and the MantaRobot scored lowest (Table 3). There were statistically significant, weak positive correlation between Pacifica system usability ratings and Eliza system usability ratings (r41 = 0.36, *P* < 0.005) and moderate positive correlation between Pacifica system usability ratings and MiRo system usability ratings (r41 = 0.44, *P* < 0.001). There was a statistically significant, weak negative correlation between MantaroBot system usability ratings and MiRo system usability ratings (r41 = −0.38, *P* < 0.005) and a moderate positive correlation between MantaroBot system usability ratings and VR system usability ratings (r41 = 0.51, *P* < 0.001).

In their free text notes, attendees considered MiRo to have “potential” and “great appeal,” however, further development was recommended in terms of a “softer feel.” The Pacifica app was “quick and easy with instant feedback” and could be used on a mobile phone “discreetly” although its accessibility for older adults was questioned. Eliza attracted mixed reviews, with some suggesting the conversational agent “lacks empathy” and that it was “hard to see usefulness in current format.” However, others suggested it “has potential” and that it was “good for communication, nice to talk to.”

Attendees envisaged applications of virtual reality to include “visit[ing] special places from childhood” and “for partially sighted people to see the ground or objects out of range.” The augmented reality application was posited as a way of reminding older adults how to complete activities of daily living such as making a cup of tea. However, attendees wondered if people living with dementia would “remember how to use the augmented reality to help them make a cup of tea.” Comments also included that “AR has potential for future use but would need to be integrated into the NHS” although confidentiality and risk were highlighted as potential barriers to use. Finally, the MantaroBot, which attracted the lowest SUS score, was found to be “clunky” and in need of a “better controller.” While some were “doubtful of real benefit,” others saw the MantaroBot as “potentially useful for discharge from hospital.” Again, the activity stimulated attendees’ imaginations, and many of the technologies they had experienced in this activity, some for the first time, were incorporated into their ideas for the final, Invention Test, activity later in the day.

#### Invention Test

Three of the six multi-perspective groups (1, 4, 5) co-created ideas which targeted the specific needs of the persona that they had considered in the first activity, while others were tangential or more general. The majority of groups also took inspiration from the technologies they had tried using earlier in the day and used these within the solutions they proposed.

Of those that directly addressed the persona in their Vignette (see Supplementary Appendix A), Group 1 (John: recently made redundant, isolated, and looking for things to occupy his time) presented a suite of solutions including use of online education groups and an online train enthusiast group to address social isolation, use of video calling and a Nintendo Wii to increase fitness and engage with family and grandchildren. Group 4 (Ashok: main carer for wife with dementia, social isolation compounded by hearing problem) suggested a person-centered technology care package consisting of technology chosen specifically for the user, and adapted to their specific needs, for example ensuring that the manuals for the technology were provided in the user’s own language. Group 5 (Julie; aging with an intellectual disability, mobility restricted through broken ankle, isolated at home) suggested that the MantaroBot could be used on an allotment, if it could be improved with solar power and be made waterproof. The aim was to support the care recipient to visit her allotment and re-engage with friends on neighboring allotments whilst she is recovering at home.

Group 2 proposed a virtual avatar to help older people who experienced depression, anxiety, or loneliness, to enable them to start new social groups. Group 3 presented a generic system they called ‘I care,’ based on principles of shared care, but in a virtual world. The idea was to provide a local forum for people in a discrete locality, whereby members could assist each other, refer each other to help and support for confidence building and other low risk activities that might replace some of the work of a support worker. The final group addressed older adults’ mental health needs in general by imagining a pair of glasses used as an augmented reality/virtual reality device, which the group titled ‘Virtual Library Live.’ The tool would allow access to downloading books, music, photographs, and videos. It could be used for a future hospital patient to review the journey into hospital and preview the experience of a certain procedure, to reduce anxiety around operations in adults and children. The same device was envisaged to be of benefit in care homes as a form of cognitive stimulation. Virtual gloves connected to the device would permit game-playing, and augmented reality elements would assist people with memory difficulties to complete activities of everyday living. This final proposal was judged the winner of the Invention Test.

### Workshop 2 Evaluation

As with the first workshop, attendees said they enjoyed sharing ideas and views and meeting new people from a range of backgrounds and professions as well as hearing the THAW Ph.D. students’ presentations. The addition of Vignettes to the THAW presentations and hands-on Scavenger Hunt in the second workshop was well-received:

“Interactive nature, case study to focus discussions, hearing about emerging research, with a desire to learn from us too… worked because of careful matching/planning by the organizers.”

Additionally, seating different stakeholders together, positively contributed to the attendee experience, with suggestions for future events to engage even more older people from different backgrounds, including Black, Asian and Minority communities.

However, it was the ability to see and use new technology that for many attendees was the most important aspect of the workshop. As one attendee said, it was

“Good to get your hands on technology”

and another, that it was:

“good to see different devices and try!”

This interaction appeared to help attendees realize the potential of using new technology with older people, as one stated it is

“Very useful to see devices that can help older people in everyday life.”

Another commented that they appreciated:

“Applying the information to devising a new product that supports good mental health and wellbeing”

### Workshop 2 Summary and Reflection

The second workshop focused on envisaging and co-creating services and products for older adults to address situations that can challenge mental health and wellbeing in later life. The program of activities eased attendees into conversations which may otherwise have been difficult to start, and naturally led to the final activity, which provided space for co-production based on the vignettes and technologies tried on the day.

Having each group work with a different vignette, permitted exploration of a range of situations that can lead older adults to be lonely, isolated or experience poor mental health. Attendees were thereby able to gain some insights into the complexity of people’s lives to develop a realistic proposal for how or where technology could play a role. In addition, many of the solutions generated in the Invention Test centered on providing older adults with ways of connecting with new groups, or even starting their own groups, using technology, demonstrating the potential of technology in this area to be well recognized.

## Discussion

As a method, the facilitated, interactive THAW workshops were successful in engaging stakeholders from multiple perspectives in open conversations about late life mental health, social isolation, and technology. The intention was to go beyond known obstacles and barriers (e.g., lack of knowledge, inability to access the necessary technology and services, and barriers presented by mental health difficulties (Greer et al., 2019) to consider how to get technology into people’s hands for long-term benefit. The first workshop confirmed the feasibility of bringing stakeholders together to discuss late life mental health and potential roles for technology. The second session demonstrated how older adults can be involved as equals in co-producing proposals for new technology services in line with the five principles of co-production: building and maintaining relationships; reciprocity; sharing of power; including all perspectives and skills and respecting and valuing the knowledge of all (Walker, 2019).

Working together as equals to develop new solutions, provides opportunities for deeper understanding of the situations and concerns of all groups. Older adults sharing their personal experiences of mental health challenges, as well as attitudes toward and interactions with services, can provide an invaluable reality-check. For example, being able to discuss why people do or do not use a particular device or service (e.g., NHS website and apps, online mindfulness) and hearing how they currently use technology to manage their mental health (e.g., self-reliance, averting loneliness, and improving mood; Andrews et al., 2019).

By using a seating plan to mix up attendees with different professional and personal backgrounds, the workshops provided a safe environment, with equal value placed on the contributions of each group member. One benefit of using vignettes in the second workshop was that they were versatile and enabled different problems to be highlighted and responses from the different groups to be compared and contrasted (Skilling and Stylianides, 2020). Vignettes have been used for a wide range of purposes and to explore a variety of issues such as the learning and development of care home staff (Flick et al., 2013), mental health disparities (Lapatin et al., 2011), educational research (Skilling and Stylianides, 2020) and researching social work values (Wilks, 2004). This study suggest that vignettes can be an important tool in coproduction. Specifically, the vignettes helped elucidate the attendee’s views, feelings, and interpretations of mental health issues during the second workshop. In this respect, the use of vignettes is situated within qualitative research methodologies and interpretative approaches aiming to elicit beliefs and understanding (Miller et al., 1997; Gourlay et al., 2014). However, the vignettes were designed to provoke participant responses which should not be mistaken as reactions to ‘actual events’ (Skilling and Stylianides, 2020). They were beneficial because they helped guide conversations and safely promoted interactions on sensitive or potentially distressing issues, and so enabled the constructive sharing of ideas between participants.

Furthermore, by ‘simulating’ life situations, the vignettes provoked discussion and offered an entry point into the complex issues of mental health. This enabled the views of attendees from different fields to be unpacked and for them to interrogate these issues together (Kandemir and Budd, 2018). The simulated nature of vignettes provides enough emotional ‘distance’ to enable attendees to contribute confidently and safely (Simon and Tierney, 2011). Three of the vignettes directly shaped and structured interactions during the Invention Test, with those groups co-producing solutions targeted at the specific needs of the personas (1, 4, 5). These groups developed person-centered solutions compared to the more generic solutions offered by other groups. Additionally, the attendees used the vignettes to speak openly and honestly about the challenges that may be presented by some of the technologies. They also felt confident to suggest non-typical uses of the technologies on show, for example, for scenario 5 (Julie) suggesting that a telepresence robot (MantaroBot) could be used outside on an allotment.

Frontline staff in health care and social care services – who are usually the ones setting up and supporting technologies for their clients – were also able to speak as equals with managers and commissioners of services. Hearing the voice of staff as experts and recognizing their needs in relation to technology adoption and support are also crucial for successful implementation and rollout of technologies as part of person-centered services (Christie and Marshall, 2019). Sharing expertise from different stakeholders, including social care and health care, meant it was possible to consider how both types of services might make use of technology to support their work with older adults, and to better understand the experiences and views of the older people they support (Astell et al., 2018).

Considering priorities for services and technologies to address the needs of older adults’ mental health and wellbeing, the activities successfully elicited thinking that bridged current gaps between (health and social care) services. Overcoming existing silos is essential for tackling the conditions that negatively impact late life mental health and wellbeing as the COVID-19 pandemic has highlighted (e.g., Steptoe and Di Gessi, 2021). The attendees were also mindful of the barriers that face many people in participating in the digital world, especially those who are socially isolated (Helsper and Reisdorf, 2017). This led to discussion about the need for Internet access, digital literacy and affordable or loanable devices for empowering people to meet their needs for social interaction and mental stimulations.

Results from the Scavenger Hunt also demonstrated that while older adults and other stakeholders foresee potential benefits from new technology, they also recognize potential barriers to their development and roll out, in addition to limitations around the applicability of some technologies requiring physical dexterity or cognitive engagement. For some attendees, this was their first opportunity to try using technologies such as virtual reality and mental health apps, providing them with an experiential learning opportunity.

The interactive methods used here also elicited engaged discussion and debate about the technologies the attendees were able to try out. In relation to late life mental health, the poor usability rating of the MantaroBot at both workshops, is interesting given the focus on telepresence robots as a potential solution for tackling loneliness and social isolation, particularly among older adults (Niemalä et al., 2019). A growing number of telepresence robots are available for purchase, and one in particular – Giraff – has been developed and used in a number of European research projects focused on supporting older adults at home e.g., Coradeschi et al., 2014). However, some attendees did see potential benefits of MantaroBot, particularly for monitoring after hospital discharge, with one team proposing that it could be part of their new co-produced service solution in the Workshop 2 Invention Test. In this activity, the groups also made clear that technology-based solutions should continue to be person-centered and acknowledged the need for support to be in place to ensure older adults can truly benefit.

These findings suggest that such discussion and hands-on interaction is important for co-producing future solutions, for example the potential of using robots. In the second workshop there was a negative perceived usability correlation from the adapted-SUS scores between MantaroBot and MiRo, indicating a weak but visible relationship between those that rated MiRo with high perceived usability and MantaroBot with low perceived usability and vice versa. This suggests that the usability of robotics can be heavily influenced by their implementation. The positive perceived usability correlation between MantaroBot and VR indicates a moderate relationship between people who rated MantaroBot and VR with high perceived usability. The problems found by attendees with the MantaroBot may have been equal to the problems identified with VR and this was reflected in the usability scores.

The successful inclusion of measures such as the adapted SUS (Bennion et al., 2020) in co-production is important, particularly for iterative projects requiring users to evaluate technology solutions over time. In the second workshop, mean SUS scores helped highlight the usability of the technologies within the target demographic, while the individual questions of the scale helped generate discussion about each technology. It was noted that some of the questions of the scale were not answered by all older adult attendees. This may have been due to the language of the scale being problematic for older adults to understand. Use of the more recent simplified SUS scale for cognitively impaired and older adults by Holden (2020) would be beneficial in future older adult co-production workshops.

Overall, both workshops were very successful but there was some learning for organizing future events. Attendees’ keenness to interact with the Scavenger Hunt technology in both workshops, meant that queues formed, and timings overran. The size of the room may be important in this respect. Similarly, in the first workshop, multiple attendees at each table wished to present back on the technologies they tried to get working, leading to this taking longer than anticipated.

In conclusion, interactive facilitated workshops can provide a method for co-producing new thinking about late life mental health and wellbeing. This includes taking a holistic view of the complexities of older adults’ lives that can lead to loneliness, social isolation and poor mental health and wellbeing. Such understanding is vital for considering how technology can be offered and incorporated into people’s lives. The findings suggest that solutions are needed that combine technologies (e.g., tablets, robots, apps) with access to Internet, support for setting up and maintaining technologies and the potential for personalization (e.g., hobbies, language, and cultural history). These outputs indicate that the methods described in this paper constituted important tools for those who wish to shape technology for mental wellbeing in later life.

## Data Availability Statement

The raw data supporting the conclusions of this article will be made available by the authors, without undue reservation.

## Ethics Statement

Ethical review and approval was not required for the study on human participants in accordance with the local legislation and institutional requirements. The patients/participants provided their written informed consent to participate in this study. Written informed consent was obtained from the individual(s) for the publication of any potentially identifiable images or data included in this article.

## Author Contributions

AA drafted the final manuscript. JA drafted sections of the manuscript about late life mental health, summarized the invention test data and provided feedback on the draft. MB summarized the Workshop 2 Scavenger Hunt, conducted the SUS analysis, and provided feedback on the draft. DC drafted sections about the vignettes and results from the evaluation surveys and provided feedback on the draft. All authors contributed to the article and approved the submitted version.

## Conflict of Interest

The authors declare that the research was conducted in the absence of any commercial or financial relationships that could be construed as a potential conflict of interest.

## Publisher’s Note

All claims expressed in this article are solely those of the authors and do not necessarily represent those of their affiliated organizations, or those of the publisher, the editors and the reviewers. Any product that may be evaluated in this article, or claim that may be made by its manufacturer, is not guaranteed or endorsed by the publisher.
