# Supplementary material for: Technology for Healthy Aging and Wellbeing: Co-producing Solutions
Source: Front Psychol. 2021 Dec 2;12:745947. doi: 10.3389/fpsyg.2021.745947 (PMC8674183; doi:10.3389/fpsyg.2021.745947)
Supplement: Supplementary file 1 [file Data_Sheet_1.PDF]

Appendix A – THAW Workshop 2 Vignettes

Six vignettes were developed presenting an individual in a situation that could lead to loneliness, social isolation or have a negative impact on mental well-being. Vignettes have been used to for a wide range of purposes and to explore a variety of issues such as the learning and development of care home staff (Flick, et al., 2013), educational research (Skilling & Stylianides 2020) and researching social work values (Wilks, 2004). They offer an entry point into these complex issues enabling participants to unpack and interrogate their views (Kandemir & Budd 2018). As such, the approach is situated within qualitative research methodologies and interpretative approaches aiming to elicit beliefs and understanding (Miller, et al., 1997; Gourlay, et al., 2014). Vignettes, however, are short narratives that are an incomplete representation of reality. They are designed to provoke participant responses which should not be mistaken as reactions to 'actual events' (Skilling & Stylianides 2020). For this workshop, vignettes acted as a vehicle to shape and structure the interactions during the day's activities. Accordingly, the approach was used to gain insights into the views, feelings and interpretations of the participants rather than assessing their 'actual' behaviour. They were judged beneficial for helping to guide conversations and safely promote interaction of sensitive or distressing issues, thus enabling the constructive sharing of ideas within the workshop environment.

The content of the vignettes drew on the collective experiences of the THAW researchers to create representations of older people. The process of creation was iterative where personal insights and professional experiences of working with older people was used along with imagined and invented aspects to ensure a diversity of representation in the scenarios. One benefit of using vignettes is that they are versatile in their presentation and application to different contexts (Skilling & Stylianides 2020). In this respect, the researchers took a variety of personal situations to develop

six vignettes for the THAW workshop (see Appendix A). This enabled different problems to be highlighted and responses from the groups to be compared and contrasted. As the stakeholders were from diverse backgrounds and experience, the terminology avoided jargon and was kept simple.

It was felt important that the vignettes should resonate with participants (Skilling & Stylianides 2020) but also offer the odd ‘curve ball’ to critically challenge participants. The vignettes, therefore, attempted to be ‘realistic’ but also ‘imaginative’, drawing on both common and unusual aspects to inform each case stud. Ethical considerations meant creating plausible scenarios but also providing ‘distance’ to enable participants to contribute confidently (Simon & Tierney 2011). Accordingly, the vignettes were written in the first person, enabling participants to draw on their personal experiences whilst avoiding personal disclosure, protecting their confidentiality and allowing discussion of sensitive and complex issues safely. Each vignette was different for each table but contained a similar amount of information and included descriptions such as age, health and family relationships and was accompanied by questions designed to gather information from the participants. Pens, paper and post-it notes were also provided.

Case Study One - John

My name is John. I am a man of 63 and live with my wife Karen. I have two grown-up daughters and three grandchildren who live locally. Recently, I was made redundant from my job as a buyer for an engineering company when it went into administration. I had worked for the company for 22 years. It was a very busy job, and I was a very committed worker. I would often work up to 60 hours a week to get things done.

At first, I was looking forward to the free time, but it has been different from what I expected. The days seem very long, and I frequently feel bored. Karen still works and is out all day, my children are very busy with their own lives and my grandchildren are at school. Sometimes I pick my grandchildren up from school but most days I have nothing to do. I watch a lot of sport on TV or spend hours on the internet researching my lifelong interest in train services and railway history.

My father died recently after a long illness. My mother is in her 80s and seems to be managing but I do worry about her. I have also been diagnosed with diabetes and my GP, Dr Hussain has told me to lose some weight and do more exercise. I have tried to do this but find it difficult. My wife has suggested I get involved in some sporting activities, but I am not sure this will help. I have asked the local leisure centre for help and a fitness instructor, Mike, has emailed me to invite me to call him.

Perspectives:

John

Karen (John's wife)

Dr Hussain (John's GP)

Mike (John's fitness instructor)

Gail (Commissioner of Services)

Gail works for the local health and social authority who are looking at ways to support mental well-being in later life using technology. She has been given a budget to look at developing new services in this area.

Tracy (ICT Developer/Company)

Tracy specialises in looking at ICT solutions. She works as a consultant for a development company and has a background in computer design and programming.

Paul (Researcher)

Paul is a research fellow for a centre for assistive technology. He is interested in how to evaluate technology to support mental health.

Case Study Two - Pam

My name is Pam. I am a 55-year old woman. My life can be a bit chaotic. I live in my flat in the inner city. I have a son from a previous relationship, but he has an alcohol problem. When I do see him, he can be abusive and shouts at me. My social worker Gary has told me I mustn't see him, but I find it hard to say 'no'.

I have epilepsy and experience regular fits leading to hospital admission. My GP, Dr Hussain, has told me I need to look after myself more and watch what I eat and drink, but my concentration and motivation are not very good. I am on benefits as I find it hard to work with my illness. I have worked in a shop but left school without any qualifications and so have found it difficult to get a job.

I tend to stay at home a lot. I have a good neighbour, Pete who pops in most days to say 'hello'. He is really into playing computer games on his mobile phone, but I cannot afford all that stuff and find it all a bit confusing. My dad is still alive but lives across the city in a residential care home. I have no transport and don't like travelling on buses in case I have a fit. I don't have many interests but do enjoy watching TV and films. I have always wanted to travel but my health and money mean I rarely leave the city.

Perspectives:

Pam

Pete (neighbour)

Dr Hussain (GP)

Gary (social worker)

Gail (Commissioner of Services)

Tracy (ICT Developer/Company)

Paul (Researcher)

Case Study Three - Jane

My name is Jane. I am 90 years old and a retired school teacher. I have recently moved into sheltered accommodation after my husband died last year. We were married for 60 years. My son Dean lives abroad and so I do not see him very often although he calls me every day on the telephone. I miss my old home where I lived with my husband. It had lots of memories and I knew the area well.

I am described as 'good for my age' but I do get very tired and sometimes forgetful. I have given up driving after being persuaded by my son it was best for me. Many of my friends have died although my best friend Sally is still alive. She speaks to me most weeks on the telephone but we don't see each other as much, as her husband is ill and she has to care for him.

I have always been very active and am still a member of the ladies bowling club. I have been thinking of stopping because I find I am forgetting the score and am embarrassed. I also used to enjoy reading but my eyesight is not so good now and so reading is not so enjoyable for me. Recently, I have been going to a computer group run by Linda from a local charity in the communal lounge of the sheltered accommodation. I have enjoyed learning about the computer but am not sure if it will be of any use to me.

<u>Perspectives:</u>

Jane

Sally (Friend)

Linda (Charity worker)

Gail (Commissioner of Services)

Tracy (ICT Developer/Company)

Paul (Researcher)

Case Study Four - Ashok

My name is Ashok and I am the main carer for my wife who has dementia. I am 75 and have been caring for my wife for 3 years. I love my wife very much and I do not want her to go into a care home because you hear so many bad things about them. I don't even like carers coming into my home as I don't quite trust they are doing a good job.

We have no children, but we do have nieces and nephews, although they do not live locally so we don't see them very often and I don't like to bother them. We live in an area where there are few ethnic minorities and so do not have much contact with neighbours or friends in the area. I do get very tired and feel isolated. I came to the UK from India in the 1970s and still have some family living there. When I first arrived in the UK I experienced a mental breakdown and had to go into hospital for a time to recover.

I have been attending a group for Asian Elders run by Bipin, which I enjoy but as my wife is becoming more unwell, I may have to stop. She sometimes feels upset when I am attending the group. My hearing has also recently deteriorated and so even watching TV or listening to the radio is getting harder. I have been seeing a consultant, Dr Sperry but he feels this may not get any better. I don't read much as I find it difficult to get written literature in my main language, Gujarati. I

have a mobile phone I use for emergencies but use no other technology. I have heard about different things you can get to help but have no one to show me or help me if it goes wrong.

<u>Perspectives:</u>

Ashok

Bipin (Group Coordinator)

Dr Sperry (Consultant)

Gail (Commissioner of Services)

Tracy (ICT Developer/Company)

Paul (Researcher)

Case Study Five - Julie

My name is Julie. I am 58. I have a learning disability. I live with my mum in a bungalow in a small village. I can look after myself but do need some help with money and making decisions about what to do. Until recently, I went to an allotment group most days but broke my ankle and cannot go at the moment. I have missed all my friends there and now I only see my mum and younger sister Nicky. Nicky helps me with my computer. I play games every day and have Facebook. I have lots of Facebook friends but not many friends where I live.

The manager of the allotment group says he is worried about me coming back to the allotment group because I cannot walk as well since I broke my ankle. I have been seeing my occupational therapist, Sophie, to see if any equipment can help me. I can get very anxious about going out now I cannot walk as well. Perhaps I need to go to a different group, but I don’t want to because I will miss my friends.

My Mum is also not very well, and I am scared about what will happen to her. I want to live with my sister Nicky if Mum dies but she says she is very busy. This makes me feel sad and worried about what will happen to me in the future. When this happens, I like to eat a lot and have recently put on lots of weight.

Perspectives:

Julie

Nicky (Sister)

Sophie (Occupational Therapist)

Gail (Commissioner of Services)

Tracy (ICT Developer/Company)

Paul (Researcher)

Case Study Six - Jean

My name is Jean. I am 82 and I have recently had a stroke. This means I have some physical weakness on my left side. After a period of rehabilitation at a local hospital, I have come home but am finding it very difficult. I live in a house on a big estate. I am very close to my daughter Valerie and her children but have always been very independent.

My husband died when I was 62 and since that time I have happily lived alone and enjoy my own company. I did use to go and stay with Valerie quite often but recently have stopped going. Since my stroke, I feel there is an evil presence in her home, and I do not like to go. At home, I also sometimes hear things in the night that scare me. My daughter has said she is very worried and at her wit's end and doesn't know how she can help. When I hear the noises, I ring Valerie using my mobile and this can be many times during the day and night.

I sometimes get so down that I no longer feel like living. I feel like I have nothing to live for and want to die. My Community Psychiatric Nurse, Carl has suggested anti-depressant medication, but this made me feel agitated. I don’t have other friends or relatives I see regularly but I would like to make contact with old friends and family although I don’t know how.

Perspectives:

Jean

Valerie (Daughter)

Carl (Community Psychiatric Nurse)

Gail (Commissioner of Services)

Tracy (ICT Developer/Company)

Paul (Researcher)
